# Conventional and Advanced Methods Used for the Diagnosis of Fascioliosis, a Food-Borne Zoonotic Disease

**DOI:** 10.1155/japr/1353367

**Published:** 2025-01-07

**Authors:** Md Haydar Ali, Md. Shahadat Hossain, Sharmin Shahid Labony, Anita Rani Dey, Joydeep Paul, Md. Abu Hadi Noor Ali Khan, Md. Abdul Alim, Anisuzzaman

**Affiliations:** ^1^Department of Parasitology, Bangladesh Agricultural University, Mymensingh, Bangladesh; ^2^Department of Pathology and Parasitology, Hajee Mohammad Danesh Science and Technology University, Dinajpur, Bangladesh; ^3^Department of Biotechnology, School of Life Science and Biotechnology, Adamas University, Kolkata, India; ^4^Department of Pathology, Bangladesh Agricultural University, Mymensingh, Bangladesh

**Keywords:** diagnostic methods, *Fasciola gigantica*, *Fasciola hepatica*, parthenogenic *Fasciola*

## Abstract

Fascioliosis is a food-borne zoonotic helminth infection caused by flatworms belonging to the family Fasciolidae, primarily affecting ruminants. The chronic form of fascioliosis is the most prevalent and is characterized by anemia, weight loss, cirrhosis, and liver dysfunction, along with atrophy, jaundice, and bottle jaw. In humans, infection results in fever, nausea, skin rashes, and severe abdominal pain. Climate changes and human-driven environmental alterations have contributed to an increasing incidence of fascioliosis in various regions. *Fasciola* species are widely distributed and have a high occurrence in tropical countries. Approximately 2.4–17 million humans are afflicted by fascioliosis in tropical and subtropical areas, with an additional 180 million facing the risk of infection. Fascioliosis poses a notable threat to ruminants; over 700 million production animals are at risk, and global annual financial losses surpass $3.2 billion. Conventional coprological methods and advanced molecular techniques are employed to diagnose fascioliosis in animals and humans. Within endemic areas, timely and accurate diagnosis is critical for successful prevention and treatment. Molecular approaches such as various PCR techniques and serological methods are extensively utilized to diagnose fascioliosis. In this review, we describe various conventional coprological and advanced DNA-based PCR techniques along with serological methods used for the screening, monitoring, and specific diagnosis of clinical and subclinical fascioliosis in humans and animals. The information accumulated in this review will be helpful for the diagnosis of fascioliosis in the field and research laboratories.

## 1. Introduction

**Figure 1:**
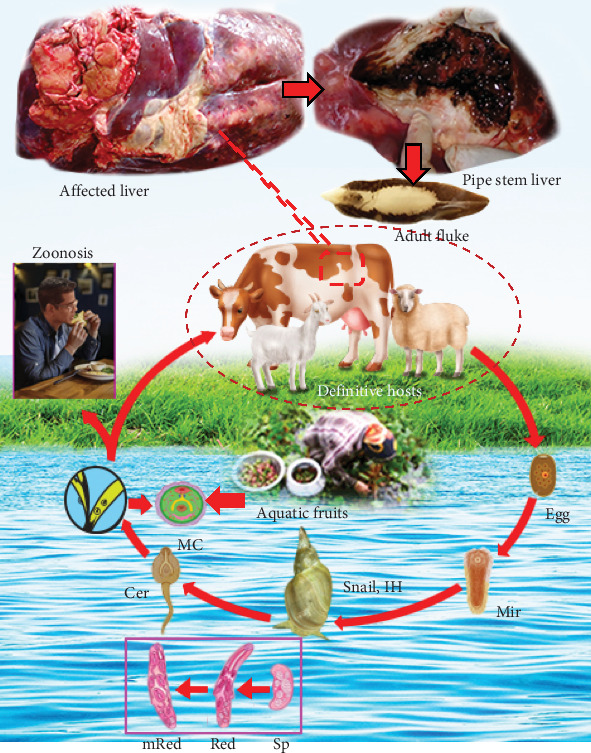
Lifecycle of *Fasciola* spp. *Fasciola* spp. are snail-borne zoonotic trematodes. Final host becomes infected by the ingestion of infective stage (MC) with contaminated food and water. They cause liver cirrhosis. Mir, miracidium; Sp, sporocysts; Red, redia; mRed, mature redia containing cercaria; Cer, cercaria; MC, metacercaria; IH, intermediate hosts.

Fascioliosis, caused by *Fasciola gigantica* and *Fasciola hepatica* (Trematoda: Fasciolidae), is a substantial menace to various animals and domesticated ruminants. The flukes primarily affect the liver and cause hepatic dysfunction, resulting in morbidity and mortality. Additionally, these parasites contribute to fertility disorders, hinder growth, and eventually decrease animal production. The infection leads to the condemnation of livers during abattoir inspections and imposes substantial losses [1, 2]. The illness manifests in acute and chronic forms, with chronic fascioliosis being more prevalent in livestock, particularly in ruminants. In ruminants, chronic cases are marked by massive liver cirrhosis, anemia, weight loss, jaundice, and eventually the development of bottle jaw and may succumb to the ailment. Conversely, acute fascioliosis is defined by hepatomegaly and the development of hemorrhagic tracts in the liver. In humans, the disease presents with fever, swollen liver, severe abdominal pain, and skin rashes [3]. Rarely, the flukes can infect subcutaneous tissue, lungs, lymph nodes, eyes, peritoneal cavity, and other organs [4]. Definitive hosts, including ruminants and humans, get infected by ingesting metacercariae (MC, the infective stage) present on grass, aquatic vegetables and fruits, and/or in water (Figure 1). Upon ingestion, MC undergoes excystation in the small intestine liberating juvenile flukes. The young flukes penetrate the duodenum, traverse inside the peritoneum, migrate toward the liver, and enter into the parenchyma. Eight to 10 weeks following infection (pi), they mature in the bile ducts and begin the process of depositing eggs, which are then eliminated in the feces [5].

The prevalence of fascioliosis is increasing day by day, which is attributed to factors like climatic variations and human-induced environmental modifications [6, 7]. Although *Fasciola* spp. are highly prevalent in tropical countries [8], *F. hepatica* is prevalent in temperate Eurasian nations such as Turkey, Austria, and Italy, as well as in American countries including Brazil, Argentina, and Mexico, where primarily taurine cattle are affected. *F. gigantica*, on the other hand, is common in Asia and Africa. The geographic range of these two parasites can intersect, particularly in the equatorial zone. There are areas where *F. gigantica* and *F. hepatica* are found together, including Egypt, Armenia, Niger, Algeria, South Africa, and Iran. The hybrid variant resulting from the interspecies cross-fertilization between *F. gigantica* and *F. hepatica* is frequently observed and referred to as the intermediate stage of *Fasciola* [8, 9]. Moreover, parthenogenic [10] *Fasciola*, a variant lacking spermatogenic capability, is prevalent in several countries, including Vietnam, South Korea, China, Thailand, Myanmar, Nepal, Bangladesh, and India. Parthenogenic *Fasciola* has not been identified in Iran, Afghanistan, Pakistan, Indonesia, Cambodia, Spain, Peru, Ecuador, Egypt, Nigeria, Algeria, and Tunisia. This kind of parthenogenic *Fasciola*, which is incapable of generating sperm, had been generated due to the hybridization of *F. hepatica* and *F. gigantica*, which occurred around 2000 years ago in China. Therefore, the subsequent generations lost their ability for spermatogenesis but gained the capacity for parthenogenesis, leading to clonal expansion and eventually becoming obligate parthenogenic *Fasciola* [11]. As a significant food- and water-borne emerging zoonosis, fascioliosis affects a range of 2.4–17 million humans living in tropical or subtropical areas [12]. In addition, over 180 million individuals and 700 million domestic animals are at risk. Only in the livestock industry, there is an estimated loss of $3.5 billion per year worldwide [8, 13].

**Figure 2:**
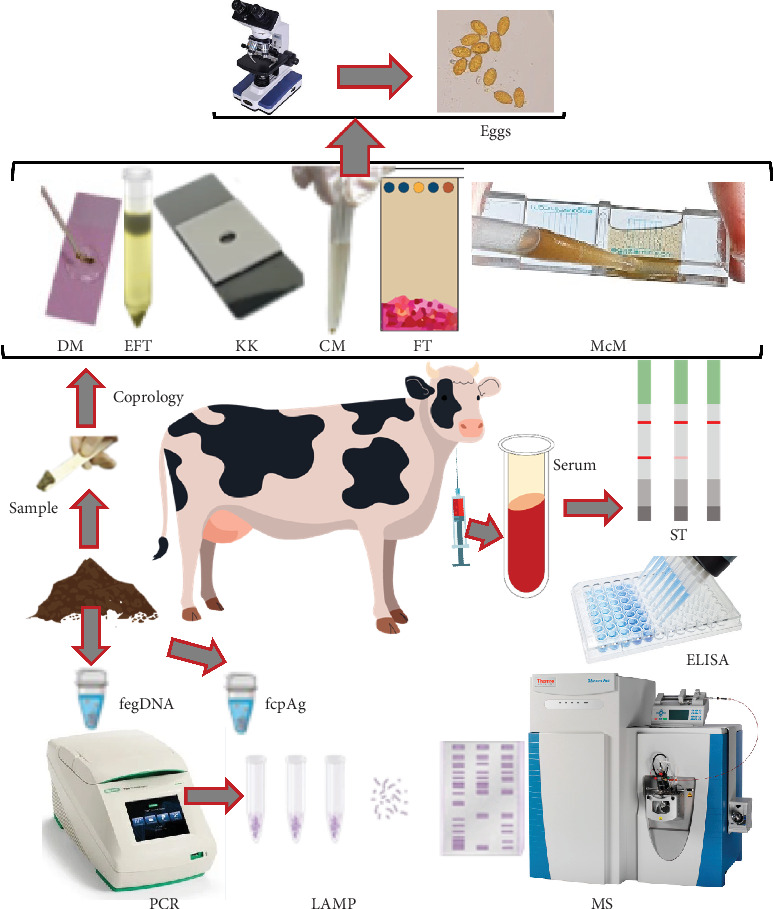
The schematic diagram shows the methods described in this manuscript. DM, direct smear; EFT, ether–formalin technique; KK, Kato–Katz; CM, centrifugation method; FT, floatation technique; McM, McMaster; ST, strip test; ELISA, enzyme-linked immunosorbent assay; fegDNA, *Fasciola* egg DNA; fcpAg, *Fasciola* coproantigen; LAMP, loop-mediated isothermal amplification; MS, mass spectrometry.

Efficient and precise diagnosis of fascioliosis plays a crucial role in mitigating its economic impacts. Diagnostic methods rely on detecting substances released by *Fasciola*, such as cell-free circulating DNA (cfDNA) [14] and metabolic antigens in the feces or blood [15] and eggs in the feces of the definitive hosts [16]. The sources of amplifiable cfDNA can be tegumental sloughing and degraded or nonviable MC ingested during grazing and/or degraded eggs [17]. Coproscopy allows for the identification of fluke eggs, but its utility is confined to the patent period (specifically from 8 to 12 weeks after infection), and liver tissues become damaged by this period. However, the conventional copromicroscopic approaches fall short in accurately identifying and distinguishing between different *Fasciola* species, as their eggs exhibit morphometric similarities [18]. Various molecular methods for detecting DNA extracted from adult *Fasciola* or their eggs have recently been employed for diagnostic purposes. These techniques have been proved valuable in distinguishing between different species of flukes [19]. Nevertheless, these techniques share a fundamental limitation with coproscopy, as they rely on amplifying DNA sourced from parasite eggs. To tackle this obstacle, specialized diagnostic tools based on coproantigens have been devised, concentrating on metabolic antigens produced by both mature and late immature flukes. Additionally, serological methods such as immunofluorescence assay (IFA), indirect hemagglutination assay (IHA), and enzyme-linked immunosorbent assay (ELISA) have been devised, capable of detecting both the initial and prolonged manifestations of the illness (Figure 2). Despite enormous impacts, the diagnostic procedures of fascioliosis have been scattered in different works of literature. The review is aimed at describing all the available conventional and molecular methods along with their limitations.

## 2. Coprological Methods

To conduct coprological examinations, stool samples should be collected directly from the rectum. If it is impossible, samples can be collected from the top of the freshly voided fecal pad. It is better to examine the samples immediately after collection. If immediate examination of the collected samples is not possible, then the samples can be preserved using 10% formalin. However, samples can also be stored in a refrigerator (4–8°C) until analysis. For coproscopy, the analysis should be conducted within 5 days of sampling, whereas the coproantigen test necessitates examination within 2 days of sample collection. Stool samples for PCR assay should be preserved at −20°C until the DNA is extracted. In the case of humans, freshly voided stools during defecation should be collected (Figure 2).

### 2.1. Coproscopy

Various coprological methods are employed for quantitative analysis, including the wet smear/direct smear technique, sedimentation, floatation, centrifugation, ether–formalin method, Kato–Katz technique, and Flukefinder method. For the quantification of egg per gram (EPG) of feces, Stoll's ova counting technique, Mini-FLOTAC, and McMaster method are often utilized. In the wet smear or direct smear method, a small amount (20–50 mg) of the sample is taken on a glass slide with a toothpick, and a thin smear is prepared by adding a drop of water for microscopic examination. However, this method is criticized for its low sensitivity, which may partially be minimized by examining the same sample, at least for triplicates [20]. In the sedimentation technique, approximately 5–10 g of feces is suspended in normal saline or phosphate-buffered saline (PBS) (100–200 mL), sieved to remove coarse materials, and left for 30 min. After discarding the supernatant, the sediments are resuspended again by adding 100–200 mL of normal saline or PBS, and the washing procedures are repeated the same way until the suspension becomes clear. Therefore, the supernatant is discarded, and the sediment is examined under a microscope [21]. This is an easy method but time-consuming and unsuitable for examining a large number of samples. The centrifugation method prepares a sample suspension using the same procedure mentioned earlier. The filtrate is then centrifuged for 5 min at 1000 × *g*, the supernatant is disposed of, and the sediment is examined using a microscope [22]. Globally, the centrifugation technique is very commonly used. It is also a very simple, easy, and rapid technique, but prolonged centrifugation at high speed may cause distortion and even disruption of eggs. In the floatation technique, approximately 5–10 g of feces is suspended in saturated salt solution (SSS) (specific gravity: 1.200), saturated sugar (Sheather's) solution (specific gravity: 1.180), zinc sulfate solution (specific gravity: 1.180–1.200), or mercuric iodide solution (specific gravity: 1.630) in a total volume of 50–100 mL and sieved. Then, the filtrate is taken into a test tube up to the brim, and a coverslip is placed on the bulging surface of the suspension on the open mouth of the test tube. The test tube is left for 30 min, and the coverslip is removed and placed on a slide. Then, the slide is examined under a microscope accordingly [20]. This is also a simple method but time-consuming. To diagnose fascioliosis, the floatation technique is not commonly used; however, the technique is used to isolate fecal contamination free highly pure eggs. The floatation technique is, again, time-consuming.

In the ether–formalin technique, a fecal sample weighing 5–10 g is suspended with roughly 9 mL of formalin (10%), and the mixture is allowed to incubate at room temperature for 30 min. Afterward, each tube is supplemented with 2–3 mL of ethyl acetate, and the combination is filtered. After tightly sealing the caps, each tube undergoes a 30-s shaking, and the cap is gently removed to release any gas. The samples are then centrifuged at 2500 × *g* for 4 min, which results in four layers within each tube (ethyl acetate, bulk debris, a long column of formalin, and sedimented ova arranged from top to bottom). The debris layer is meticulously eliminated with an aspirator, and everything in the tube, except for the sedimented ova, is discarded. A drop from the resultant sediment is examined under a microscope [21]. The ether–formalin technique effectively isolates fecal contamination-free eggs from fecal samples.

In the Kato–Katz method, approximately 10–15 g of fecal sample is taken on a petri dish/paper, and a wire screen is placed on it and pressed. A cardboard is placed on a clean slide, and the hole of the cardboard is filled with feces passed out through the wire screen, and the cardboard is discarded. Then, a malachite green–soaked cellophane is placed on the smear and examined within 30–60 min [23]. The Kato–Katz method is widely used in human hospitals and diagnostic centers.

The commercially available Flukefinder kit consists of a unit comprising two sieves with mesh dimensions of around 125 and 30 nm (precise measurements proprietary), each 2 in. wide. The procedure involves mixing 2 g of feces with water and liquid soap, pouring the mixture into the Flukefinder unit, and rinsing it thoroughly with water. Then, the kit is allowed to settle for 2 min, and the supernatant is poured off. The leftover material is examined under a dissecting microscope with a 10 × magnification by adding three drops of methylene blue [24].

Stoll's ova counting method thoroughly mixes 3 g of feces with 42 mL of 0.1 N NaOH solution in a small container. Subsequently, the suspension is sieved, and 0.15 mL of the filtrate is taken using a pipette and transferred to a clean glass slide, which is then covered with a cover slip. The final step includes the examination of the slide under a microscope [16]. Each sample is examined at least in triplicate. Then, the average number of each sample is calculated, and EPG is estimated simply by multiplying the average number of eggs with 100. The method is very simple and useful for calculating EPG in helminth infections caused by trematodes. The method is highly criticized for counting errors if a plain cover slip is used. However, the problem can be minimized by using a gridded glass coverslip [19].

In the McMaster technique, fecal samples are blended with a SSS at 10:1 (45 mL of SSS + 5 g of feces), and then, the suspension is strained through a 150-*μ*m mesh sieve. Then, the suspension is loaded into a conventional McMaster chamber and left for 5 min. Therefore, each chamber of the McMaster slide is examined under a microscope using low power magnification (objective 10 and eyepiece 6–10) [25]. In the McMaster technique, EPG is estimated using the following formula:

$$\begin{array}{cc} \; & \text{EPG}=\frac{\text{number of eggs in two chambers}}{0.3}\times \text{df},\\ \text{dilution factor}\left(\text{df}\right)=\frac{\text{total volume of suspension }\left(\text{mL}\right)}{\text{used fecal sample }\left(\text{g}\right)}. \end{array}$$

The McMaster technique is not routinely used to diagnose fascioliosis; however, it can quantify the load of Fasciola infection by estimating EPG. The technique is also used to determine the efficacy of anthelmintics during the fecal egg reduction test (FERT) [26].

The Mini-FLOTAC, similar to McMaster, by which many feces can be examined, is becoming popular due to its simplicity, accuracy, and capability to detect eggs of diverse helminth species. Mini-FLOTAC is also based on the floatation of eggs in the kit chamber. However, the floatation chamber is very large and can accommodate 1 mL of suspension in each chamber; thus, the technique allows the examination of 2 mL of suspension at a time. In this technique, 3 g of fecal sample is mixed with 27 mL of SSS (dilution ratio 1:10) and sieved. Then, the chambers of the slide are loaded with the filtrate. After 10 min, the chambers are examined under a microscope using low power magnification (× 10), and eggs within the grid areas are counted [27].

EPG is estimated by the following formula:

$$\begin{array}{cc} \; & \text{EPG}=\frac{\text{total number of eggs in two chambers}}{\text{total volume of two chambers }\left(2\text{ mL}\right)}\times \text{df},\\ \text{df}=\frac{\text{total volume of suspension }\left(\text{mL}\right)}{\text{total amount of feces used }\left(\text{g}\right)}. \end{array}$$

### 2.2. Coproantigen

An adult *Fasciola* has the remarkable ability to endure for over a decade within its final host [28]. During their stay in the final host, these parasites release numerous bioactive molecules (BAMs), called excretory and secretory (E/S) products [29]. The E/S products from parasites trigger diverse immunological responses in the host. Antibodies generated against these BAMs can offer protection or serve as valuable tools for serodiagnosing fluke infections [15]. Additionally, antigens present in the E/S products can be detected in feces. Over the past few decades, various capture ELISA techniques have been proposed to identify these coproantigens [30]. However, up to now, only the in-house MM3-COPRO ELISA and the commercially available BIO K 201 kit (BIO-X Diagnostics, Rochefort, Belgium) have undergone thorough testing [31]. Both tests have been widely adopted and are now recognized as valuable tools for the precise diagnosis of early illnesses in humans and ruminants [19]. The techniques have also been proven to be instrumental in monitoring the effectiveness of flukicide treatments [32].

### 2.3. Copro-PCR

Despite residing in the gall bladder and bile duct of final hosts, *Fasciola* spp. release their eggs through feces [33]. Numerous efforts have been made to detect DNA materials in these eggs through copro-PCR. In infected fecal samples, eggs are recovered using a modified floatation technique [22]. The retrieved eggs are then resuspended in PBS and disrupted by adding glass beads, 1% SDS (sodium dodecyl sulfate), and proteinase K, followed by several freezing and thawing cycles. Subsequently, the mixture is centrifuged for 15 min at 12,400 × *g*. Then, the genomic DNA is isolated using either the phenol–chloroform method or a commercially available kit [21]. In addition, genomic DNA extraction kits are available by which DNA from ova present in freshly collected unprocessed fecal samples can easily be extracted and used for PCR [34].

For PCR-based confirmation of *Fasciola* spp., various primer sets and protocols are utilized. However, using a primer set, a 618-bp segment of the 28S rRNA gene unique to the *Fasciola* species is amplified (sense: 5⁣′-ACG TGA TTA CCC GCT GAA CT-3⁣′ and antisense: 5⁣′-CTG AGA AAG TGC ACT GAC AAG-3⁣′) [35]. In that case, a 25-*μ*L mixture including 2 *μ*L of diluted (1:30) genomic DNA, 1.5 U of Taq DNA polymerase (Fermentas, Germany), 50 mM of each dNTP (deoxynucleotide triphosphate) (CinnaGen, Iran), 2 mM of MgCl_2_, 2.5 *μ*L of PCR buffer (10 ×), and 0.2 *μ*M of each primer are used for the PCR reaction. The PCR thermal cycle consists of a 3-min initial denaturation at 94°C. The following 30 cycles consist of 30 s at 94°C, 30 s at 60°C, and 60 s at 72°C. The last phase is an extension at 72°C for 5 min. Following that, a gel documentation system is utilized to analyze the PCR products using 1.5% (*w*/*v*) agarose gel electrophoresis [36] In addition, ITS1-based PCR (forward: TTGCGCTGATTACGTCCCTG; reverse: TTGGCTGCGCTCTTCATCGAC) has also been developed, which can confirm *Fasciola* spp. [37]. The patterns derived from the internal transcribed spacer (ITS)–based PCR-RFLP (polymerase chain reaction–restriction fragment length polymorphism) [38] or pepck (phosphoenolpyruvate carboxykinase)/pold (polymerase delta)–based multiplex PCR analysis with specific primers have been developed to identify *F. hepatica*, *F. gigantica,* and hybrid *Fasciola* flukes [39].

### 2.4. LAMP (Loop-Mediated Isothermal Amplification) Assay

LAMP is a single-tube but highly sensitive molecular technique for the amplification of DNA. It was first invented in 2000 at the University of Tokyo, Japan, as a low-cost alternative to PCR to detect field-level infectious diseases. LAMP is carried out at a constant temperature (60–65°C) with four to six primers (forward primer, F3; backward primer, B3; forward inner primer, FIP; backward inner primer, BIP; loop forward primer, Loop F; loop backward primer, Loop B), recognizing six to eight distinct regions of target DNA [40].

The main advantage of this technique is it does not require a thermal cycler and is cost-effective. Reverse transcription loop-mediated isothermal amplification (RT-LAMP) combines LAMP with a reverse transcription step that enables the detection of RNA. For the LAMP-based approach, primers aimed at the ribosomal intergenic spacer (*igs*) region of the *F. hepatica* genome are utilized [41]. A total of 25 *μ*L reaction mixture is used for the LAMP assay. It contains 1 *μ*L of template DNA, 40 pmol of primers FIP and BIP, 20 pmol of primers LF and LB, 5 pmol of primers F3 and B3, 8 U of Bst DNA polymerase (New England Biolabs), 1.4 mM dNTP and 2 × reaction buffer (1.6 M betaine) (Sigma-Aldrich), 40 mM Tris–HCl (pH 8.8), 20 mM KCl, 20 mM (NH_4_)_2_SO_4_, 16 mM MgSO_4_, and 0.2% Tween 20. In this assay, a reaction mixture without the template DNA serves as a negative control. The reaction mixture is heated at 60–65°C for 30 min to 1 h. After heating, samples are kept at room temperature/4°C to observe turbidity or color change in the reaction tube. Turbidity of magnesium pyrophosphate developed in positive samples in the LAMP, which the naked eye can detect. Alternatively, color changes can be measured in the reaction tubes, which showed yellow for the positive sample, while negative samples remained pink. However, if 1 *μ*L of SYBR Green I is applied at a 1/10 dilution (Invitrogen lot number 49743A) to each reaction tube, then the fluorescence signals of the reaction mixtures can be monitored using a UV image system (UVItec). To prevent carryover contamination with LAMP, the reaction tubes are centrifuged at 1300 × *g* for 3 min and chilled at −20°C for 10 min before adding the SYBR Green I [21].

### 2.5. RFLP Analysis and Multiplex PCR

RFLP-PCR leverages the presence of distinct restriction sites in gene sequences, which can differ across various species. In this case, genomic DNA isolated from flukes, eggs, or MC is analyzed by PCR following the abovementioned procedures [36]. Then, the PCR amplicons are incubated in a total volume of 15 *μ*L by adding 1.5 *μ*L (7.5 unit/mL) of *AvaII* (Roche, Basel, Switzerland) at 37°C for 1 h. Following incubation, the amplified DNA of *F. hepatica* and *F. gigantica* will be digested by the restriction enzyme *Ava II*, and three anticipated restriction fragment sizes for *F. hepatica* (529, 62, and 27 bps) and *F. gigantica* (322, 269, and 27 bps) will be obtained [42].

Furthermore, genome-wide sequencing of different forms of *Fasciola* has demonstrated single nucleotide polymorphism (SNP) in DNA *pold*. Now, *pold* gene targeted RFLP-PCR and nuclear *pepck*–based multiplex PCR is utilized to confirm *F. gigantica*, *F. hepatica*, hybrid *Fasciola*, and parthenogenic *Fasciola*. For both species of flukes, a specific primer set (forward: 5⁣′-GAR ATG GCN MGN GTN ACN GGN GT-3⁣′ and reverse: 5⁣′-TTN CCN ACR TGN GCN CCN GTR AAN CC-3⁣′) targeting *pold* produces an amplicon with a size of 708 bp. The restriction enzyme *Alu1* digests the amplicon into 544 and 164 bp in *F. gigantica*; however, in *F. hepatica*, the PCR product remains undigested, and in parthenogenic fluke, both digested and undigested amplicons of the fluke species are evident. However, ITS1 is also used for RFLP to distinguish the species [43].

To differentiate *F. gigantica*, *F. hepatica*, and parthenogenic *Fasciola*, the pepck-based multiplex PCR is conducted using the primers (Fh-Pepck-F: GAT TGC ACC GTT AGG TTA GC, Fg-Pepck-F: AA AGT TTC TAT CCC GAA CGA AG, and Fcmn-Pepck-R: CGA AAA TTA TGG CAT CAA TGG G). The PCR reaction was conducted in a total volume of 25 *μ*L which contained 12.5 *μ*L of master mix, 1 *μ*L of 10 pmol of each forward primer (Fh-pepck-F and Fg-pepck-F), 2 *μ*L of 10 pmol of common reverse primer (Fcmn-pepck-R), and 200 ng of gDNA. Multiplex PCR reaction cycles for *pepck* consist of an initial denaturation at 94°C for 1.5 min, followed by 30 cycles at 94°C for 30 s, 61°C for 30 s, and 72°C for 1 min with a final extension at 72°C for 10 min [44]. PCR product was electrophoresed in 1.5% agarose gel and visualized by ethidium bromide [45]. In this multiplex PCR, a single band at ~510 bp is produced for *F. gigantica*, at ~240 bp for *F. hepatica*, but two bands (~510 and ~240 bp) for parthenogenic *Fasciola* [44].

On the other hand, very recently, SNP in carboxylesterase B (*Cestb*) has been found to be linked to triclabendazole (TCBZ) resistance [46, 47]. In this case, a TD (touch down)–PCR needs to be conducted using primers (forward: FExon1CestB 5⁣′-CGG GTC CAA GCA AGG ATG AG-3⁣′; reverse: RExon1CestB 5⁣′-CTC TCC TCC GAC CAT CAA ATT C-3⁣′) in a total volume of 25 *μ*L 12.5 *μ*L of master mixture (OneTaq Quick Load Polymerase, BioLab, United States), 1 *μ*L of 10 pmol of each primer, and 50 ng of gDNA. The thermal cycle consists of 3 min of denaturation at 95°C followed by 10 cycles at 94°C for 15 s, 65°C for 30 s, and 72°C for 30 s and programed to subtract 1°C from each cycle to the annealing step followed by 15 cycles of 93°C denaturation for 30 s, 60°C annealing for 30 s and 72°C extension for 40 s, and a final extension step of 72°C for 5 min. Then, the PCR product is sequenced and SNPs are detected by using bioinformatic tools. SNPs at positions 440, 643, and 788 and single amino acid polymorphisms (SAAPs) at positions 147 (R147K), 215 (E215K), and 263 (R2663K) are linked to the TCBZ resistance [46, 47].

## 3. MALDI-TOF (Matrix-Assisted Laser/Desorption Ionization Time-of-Flight) Mass Spectrometry (MS)

Current diagnostic methods for human and veterinary fascioliosis primarily involve morphological analyses [48], molecular techniques like PCR, and sequencing [41, 49, 50]. However, these methods face challenges such as the lack of standardized morphological identification, high costs associated with molecular approaches, and limited availability of PCR-based testing outside specialized laboratories. While morphological identification is quick and affordable, its accuracy decreases outside of endemic regions, and a particular difficulty is the dwindling parasitological knowledge among lab professionals.

To address these limitations of different techniques, there is a need for a more accurate, rapid, cost-effective, and comprehensible diagnostic method for identifying parasites. Over the last 10 years, clinical sample diagnosis by MALDI-TOF MS has been a common practice [51–53]. The potential of MALDI-TOF MS for identifying ticks [54], mosquitoes [55, 56], and to a lesser extent protozoa and helminths has been investigated recently in 2019 [57]. In a noteworthy development, MALDI-TOF MS was employed to identify liver flukes that are therapeutically significant based on species. Analysis of 78 *Fasciola* samples using a previously developed database demonstrated high accuracy, with 98.7% (*n* = 74) and 100% (*n* = 3) correct identification of *F. gigantica* and *F. hepatica*, respectively [58]. This demonstrates the possible usefulness of MALDI-TOF MS as a quick, affordable, and exact instrument for identifying particular liver fluke species, perhaps overcoming the drawbacks of existing diagnostic techniques.

## 4. Serological/Immunological Methods

Since *Fasciola* spp. provoke immune responses within the host body, antibodies especially IgG-based different serological techniques are used or attempted to diagnose fascioliosis.

### 4.1. Immunochromatographic (IC) Strip Test

*F. gigantica* produces cathepsin L1, which is a cysteine protease produced by cecal epithelial cells of the fluke and is released as E/S products [59], and the molecule has been named as FgCatL1. Both the recombinant pro-mature (rproFgCatL1) and mature (rmFgCatL1) proteins are capable of inducing IgG (IgG1 and IgG2*α*) in mice. An IC strip test based on anti-rFgCatL1 has been developed as a diagnostic tool. The samples are applied to the sample pads. Then, the colloidal gold-purified anti-rFgCatL1 conjugate is allowed to react with the samples. The combination is chromatographically passed across the nitrocellulose (NC) membrane by the capillary action. After 3–5 min, a positive test shows two red dots for the test and control zones, while a negative test shows only one red dot in the control region [60].

### 4.2. ELISA Test

ELISA is employed to detect either the antigen secreted by the fluke or the antibodies developed against BAMs, and the method has been named ES ELISA. To diagnose fascioliosis, 0.5 mg/mL of ES antigen in 0.05 M carbonate–bicarbonate buffer (pH 9.6) was coated on a 96-well microplate. The wells are then blocked with 2% skimmed milk. The bound ES antigens are incubated with serum samples (test samples, a positive and negative control sample). The next step is to detect antibodies ligated with E/S products using a rabbit anti-bovine IgG coupled to horseradish peroxidase (Jackson ImmunoResearch Laboratories, United States) [22].

In addition, immunodiagnostic tools have a significant advantage in detecting parasitic BAMs secreted with various matrices such as milk and urine. In dairy farming, testing bulk tanks and individual milk samples is possible if milk-based ELISA develops, and in that case, individual animal sampling is not required. An analogous approach has been suggested for beef cattle, wherein muscle liquefaction, sometimes known as “meat juice,” is acquired from the abattoir and utilized as a matrix for the ELISA of *F. hepatica* [61].

### 4.3. Western Blot

This method involves separating the fluke E/S antigens using SDS-PAGE and then using a blotting machine to transfer the separated antigens onto a NC sheet with a 0.2-mm pore size (Sigma-Aldrich). Following cutting into strips, the NC membranes are cleaned with PBST (phosphate-buffered saline with Tween 20) (0.05%) and blocked using 1% skimmed milk before being subjected to testing serum samples collected from the targeted animals. If present in the serum, the antibodies bind to the immobilized antigens, which are then detected using anti-IgG conjugated with alkaline phosphatase [62].

## 5. Conclusions and Future Perspectives

Our present knowledge about the taxonomy and epidemiology of *Fasciola* spp. is mainly based on morphological and observational research. However, the complete diversity of *Fasciola* spp. cannot be fully gained by conventional techniques employed for identifying and classifying *Fasciola*. Serological methods present themselves as crucial diagnostic tools for the swift identification of fascioliosis and the seroepidemiological screening of animals, contributing to disease monitoring and control. However, during the past 20 years, research in molecular genetics has significantly enhanced our comprehension of the genetics and taxonomy of the species of *Fasciola*, resulting in the development of complex techniques for the precise identification and distinction of *Fasciola* species. Molecular techniques have made it possible to identify the hybrid known as “intermediate *Fasciola*.” Adoption of high-throughput molecular techniques, such as next-generation sequencing, transcriptomics, proteomics, and large-scale analysis of SNPs, is necessary in analytical procedures. With the right implementation, these and other methods should shed further insight into the evolutionary processes and genetic makeup of the *Fasciola* population. More investigation into these exciting fields of study will increase our understanding of the complex biology of various *Fasciola* species, which will help create new therapeutic and immunological approaches. The development of user-friendly, highly sensitive, and very specific point of care (PoC) diagnostic tools will greatly minimize economic losses, particularly in the hyperendemic areas of fascioliosis.

## Data Availability

Data in the review article are available on request to authors.
